# Nanomedicine as a strategy to fight thrombotic diseases

**DOI:** 10.4155/fso.15.46

**Published:** 2015-11-01

**Authors:** Mariana Varna, Maya Juenet, Richard Bayles, Mikael Mazighi, Cédric Chauvierre, Didier Letourneur

**Affiliations:** 1 Inserm, U1148, Cardiovascular Bio-Engineering, X. Bichat Hospital, 75018, Paris, France; 2 Institut Galilée, Université Paris 13, Sorbonne Paris Cité, 93430, Villetaneuse, France; 3 AP-HP, Lariboisière Hospital, 75010, Paris, France

**Keywords:** animal models, drug delivery, ischemic heart, microbubbles, nanocarriers, stroke, thrombolytic

## Abstract

This review highlights the preclinical and clinical research based on the use of nano- and micro-carriers in thrombolytic drug delivery. Ischemic heart and stroke caused by thrombosis are the main causes of death in the world. Because of their inactivation in the blood, high doses of thrombolytics are administered to patients, increasing the risk of intracranial hemorrhage. Preclinical research conducted with lipid, polymer or magnetic nanoparticles loaded with thrombolytic drugs showed an enhancement of thrombolysis and a reduction of undesirable side effects. Targeted nanocarriers exhibited an increased accumulation into clot. Clinical trials were already conducted with lipid-based microbubbles combined with ultrasound and thrombolytic drug and showed thrombolysis improvement. Future validation of nanosystems is awaited in clinic. This research opens new strategies for the management of thrombotic diseases.

## Abstract (lay abstract)

To dissolve a thrombus, thrombolytic drugs are administered, but they are rapidly inactivated in the blood. High amounts are thus injected to patients with the risk to develop intracranial hemorrhages. Nanocarriers and microbubbles have been tested in preclinical models to deliver thrombolytic drugs. These systems have the advantage to protect the drug from the degradation. In clinical trials, galactose and lipid-based microbubbles associated to ultrasound and thrombolytic drugs showed an enhancement of thrombolysis. Other systems are also expected with new drugs combined or not with endovascular intervention to treat ischemic heart or stroke.

**Figure 1.**
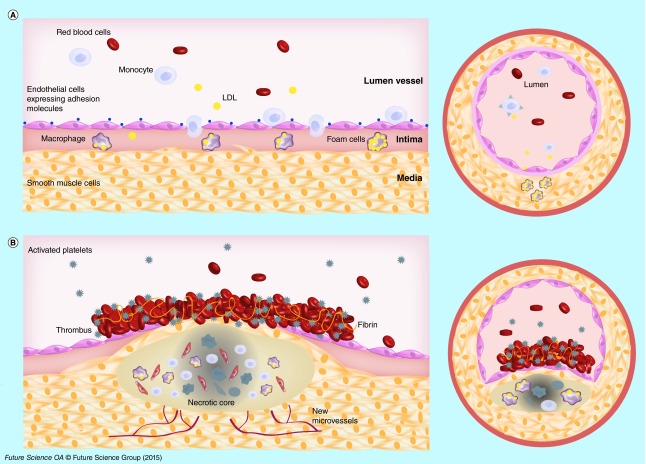
**Atherosclerotic plaque development.** **(A)** Cholesterol derived low-density lipoproteins extravasate in the intima where they are oxidized low-density lipoproteins. Endothelial cells are activated and express specific adhesion molecules. These phenomena drive the recruitment of monocytes which differentiate into macrophages expressing scavenger receptors, and the uptake oxidized low-density lipoproteins. Smooth muscle cells migrate into the intima, proliferate and contribute to foam cell formation. **(B)** In advanced stages, a fibrous cap made of smooth muscle cells and collagen fibers is formed. Apoptotic events and necrotic zones appear in a hypoxic environment inducing new microvessel development. With plaque rupture thrombogenic substances are released into the circulation and promote platelet activation and adhesion to endothelium and thrombus formation.

**Figure 2.**
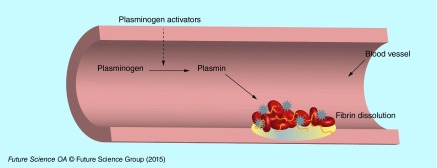
**Schematic representation of fibrin clot thrombolysis induced by thrombolytic agents in blood vessels.**

**Figure 3.**
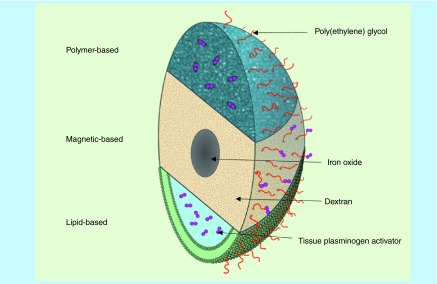
**Schematic representation of polymer-, magnetic- and lipid-based stealth nanocarriers loaded with thrombolytic drugs.**

**Figure 4.**
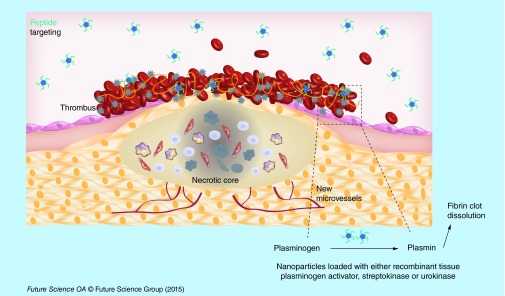
**Targeting modality by peptides.** Nanocarriers loaded with thrombolytic drugs and decorated with RGD peptides that recognize GPIIb/IIIa receptor at the surface of activated platelets, show an accumulation in the thrombus and leading to an enhancement of thrombus lysis.

**Figure 5.**
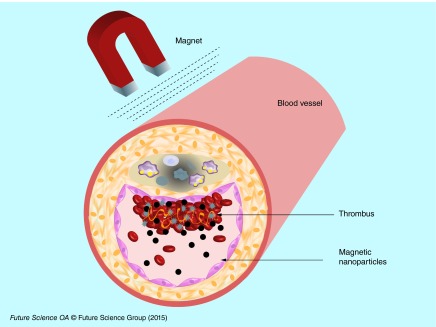
**Targeting modality by magnet.** Ultrasmall paramagnetic iron oxide nanocarriers loaded with thrombolytic drugs are concentrated by an external magnet in the thrombus to enhance its lysis.

**Figure 6.**
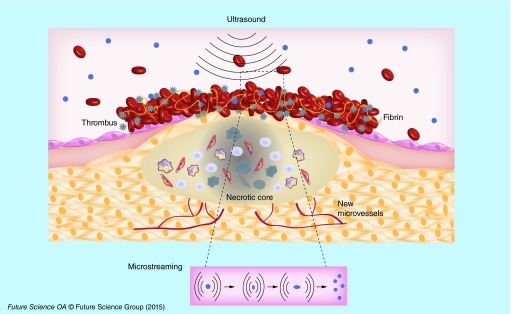
**Schematic representation of thrombus dissolution by ultrasound.**

Atherosclerosis is a multifactorial and slowly progressing pathophysiological disease. It is responsible for 17.3 million deaths per year. Among these, myocardial infarction and ischemic stroke are, and will remain the principal cause of death in the world [1]. The development of atherosclerosis is linked to some risk factors among which diabetes, hypertension, smoking, high total cholesterol, high BMI and physical inactivity [1].

The stages of this disease are now understood in detail. Atherosclerotic lesions begin as fatty streaks in lesion-prone areas in aortic bifurcations. These regions exposed to a disturbed flow may develop an activated endothelium. Low-density lipoproteins (LDLs) derived cholesterol extravasate through the defective endothelium into the subendothelial space. There, LDLs are oxidized (oxLDLs) by enzymes such as myeloperoxidase, 15-lipoxygenase or nitric oxide synthase. The recruitment of monocytes is stimulated in part by oxLDLs and is regulated by adhesion molecules expressed on the surface of endothelial cells (VCAM-1, ICAM-1). Monocytes are subsequently differentiated into macrophages (Figure 1A). The macrophages express scavenger receptors (SR-A and CD36) which recognize oxLDLs. In an advanced lesion, smooth muscle cells (SMCs) present in media, proliferate, express scavenger receptors and can also take up oxLDLs contributing to foam cell formation. SMCs also synthesize extracellular matrix proteins leading to fibrous cap development. During the progression of atherosclerosis, endothelial cells, macrophages and smooth muscle cells die by apoptosis or necrosis contributing to necrotic core formation within the plaque [2,3]. Focal calcifications, neovascularization and intraplaque hemorrhages characterize high-risk plaques with a thin fibrous cap [4]. The development of atherosclerosis begins in childhood. Sometimes, growing plaques became suddenly complicated and could break inducing a luminal thrombosis (Figure 1B). Some factors have been identified to cause plaques rupture. It is recognized the key role of the thickness of the fibrous cap and intraplaque hemorrhages induced by neovascularization. Plaque rupture exposes thrombogenic substances of the plaque to the circulating blood, promoting platelet activation and adhesion to endothelium and thrombus formation [5]. The thrombus development is initiated by tissue factor, and culminates with the circulating platelet recruitment with concomitant generation of thrombin and fibrin [6]. The thrombus is composed of fibrin monomers cross-linked through lysine side chains [5].

Atherosclerosis alone is rarely fatal, but the complications induced by thrombosis, myocardial ischemia and stroke, are the most common causes of death in western societies [3]. Arterial thrombosis differs from venous thrombosis and is therefore treated in a different way. The arterial thrombi formed after plaque rupture are rich in activated platelets, while thrombi formed in veins are rather rich in fibrin and trapped red blood cells [7]. Arterial thrombosis is thus treated with drugs targeting platelets whereas venous thrombosis is treated with antithrombotic drugs.

## Therapeutic drugs & side effects

Thrombolytic agents are able to induce thrombus lysis by the degradation of fibrin contained in clots [8]. Clot dissolution (fibrinolysis or thrombolysis) is enzymatically driven by a serine protease named plasmin, which is obtained from plasminogen. The conversion of plasminogen into plasmin is made by tissue plasminogen activators (tPAs). There are two groups of thrombolytic drugs: those which are able to bind both free circulating and/or clot bound plasminogen, and those which bind only clot bound plasminogen (Figure 2). Few plasminogen activators [5,9,10] have been approved by the US FDA for clinical applications: urokinase (UK), streptokinase (SK), alteplase (tPA), tenecteplase (TNK-tPA), reteplase (rPA) (Table 1). Natural inhibitors of plasminogen activators have been described such as α-2-antiplasmine, α-2-macroglobuline, anti-C1 esterase, α-1 antitrypsin and PAI-1 [11,12]. Because of their relatively short half-life, high quantities of thrombolytic drugs need to be administered into patients. After being administered to patients, the drugs undergo cell metabolism and are distributed throughout the body. In order to obtain a therapeutic effect, high doses have to be injected, which may lead to undesirable risk of hemorrhagic transformation after thrombolytic therapy [8]. On one hand, the degradation of the components of neurovascular unit (endothelial cells, basal membrane, perivascular astrocytes and neurons) induced by ischemia leads to the passage of fluids from intravascular space into the brain, with formation of edema and intracranial hemorrhages. In these conditions, tPA can also pass the blood–brain barrier and through interaction with the NMDA-type-glutamate receptor can potentially amplify excitotoxic calcium currents. On the other hand, indirect upregulation by tPA of MMP9 activity, which degrades extracellular matrix integrity, increases the risk of neurovascular cell death and blood–brain barrier disruption [13].

The development of new formulation of these drugs gains more and more interest. A promising strategy consists of the use of nano- and micro-carriers [14,15]. The most important advantages of these platforms are the prevention of the degradation of the drugs by enzymes in the blood and the possibility to target a thrombus using specific ligands leading to increase the drug amount into the clot. In the next section we detail the different categories of nano- and micro-carriers used to deliver thrombolytic drugs.

## Composition of nano- and micro-carriers used in thrombolytic therapy

Several types of materials, polymeric, lipid or metallic based, can be employed to deliver thrombolytic drugs (Figure 3). These materials are well-tolerated *in vivo*, possess low toxicity, and are easy to biofunctionalize. Depending on their composition, hydrophobic or hydrophilic drugs can be incorporated or attached. The formulations of nano- and micro-carriers include spheres, capsules and vesicles.

Some factors such as the size, the surface charge and the presence or not of a polymer coating, affect the clearance and the biodistribution of nano- and micro-carriers. The size can be modulated during the synthesis of nanoparticles. The charge and the composition of the surface determine the clearance by the monocyte macrophage system (MPS). Following intravenous administration, the naked nano- and micro-carriers are cleared by the MPS and the recognition is induced by the attachment on the surface of the carriers of plasma proteins named opsonins (C3b, iC3, IgG, IgM) [16]. To avoid this and to prolong the circulation time in the body, poly(ethylene glycol) (PEG), a hydrophilic biocompatible and biodegradable polymer, has been commonly used in the coating. PEG is approved by the FDA for clinical use. PEG, either adsorbed or covalently attached to the surface of nano- and micro-carriers, induces steric inhibition of opsonins and thus their attachment at the surface of the carrier [17]. Moreover PEG is easy to functionalize. Another advantage provided by the coating of nano- and micro-carriers with PEG is the possibility to attach different ligands on the surface of the carriers, in order to target a tissue or cells of interest [18,19].

### Lipid-based nanoparticles

Among the lipid-based, only liposomes and some types of microbubbles have been used in thrombo-therapy. Described 50 years ago by Bangham, liposomes represent the most used carriers to deliver active drugs. Liposomes are defined as bilayer phospholipid vesicles, mimicking the cellular membrane, enclosing an aqueous core. Since their discovery the preparation methods were diversified [20]. The choice of the method and subsequent processing steps are important because it determines the loading efficacy as well as the size. The lack of toxicity of liposomes and their biodegradation represent a significant advantage in their use as carriers to deliver thrombolytic drugs [21]. However, one of the limitations of liposomes is their poor stability in the blood flow.

### Polymer-based carriers

Synthetic and natural polymers are used in the design of nano- and micro-carriers charged with thrombolytic drugs. The polymers have the advantage of being more resistant to mechanical constraints than lipid-based ones. In addition to their biocompatibility and biodegradability, polymer carriers can be tuned in terms of size, porosity and hydrophobicity. Polymers are either functionalized with reactive groups, such as primary amines, for reaction with the drug further to the nanoparticle synthesis or are designed to directly interact with the therapeutic agent during the nanoparticle formulation. The first strategy leads to systems where the drug is charged at the surface of the particle. It has especially been applied to magnetic particles coated with a polymer shell (Figure 3). The most common is dextran which is a polysaccharide composed of α-d-glycose units that bind to each other through glycosidic bonds. Because of its affinity for iron, dextran is largely used in the coating of iron oxide nanoparticles [22]. With this strategy, the drug covalent binding is often achieved via a functionalized PEG spacer. The second method results in drug encapsulation and has been set up with different polymer types. For example, polyvinyl alcohol (PVA), a biodegradable synthetic polymer, has been formulated into a porous material to encapsulate the drug. Poly(lactic-*co*-glycolic acid) (PLGA), an FDA and EMA-approved polymer, is another promising candidate for drug encapsulation. It is largely used in medical research because of its good biodegradability and biocompatibility.

Another mechanism of nanoparticle formation is the ionic gelation process. Chitosan nanoparticles are obtained by this method allowing for drug entrapment during the assembly process. Chitosan is a natural water soluble polysaccharide. It possesses cationic and hydrophilic properties as well as good biodegradability and biocompatibility, low toxicity and immunogenicity [23]. In some cases drug retention is enhanced by ionic interaction between the drug and the polymer chains. Gelatin, a natural polymer that shows the advantage to be biodegradable and easily tunable have been modified for an enhanced interaction [24].

### Inorganic nanocarriers

Inorganic nanocarriers used in the delivery of thrombolytic drugs are principally represented by magnetic nanoparticles. They are composed of a paramagnetic iron oxide core, made of magnetite (Fe_3_O_4_) or maghemite (γ-Fe_2_O_3_) or a mixture of both, surrounded by a shell made of polymers, to improve colloidal stability (Figure 3). Depending on the size, magnetic nanoparticles are divided into two categories: ultrasmall superparamagnetic iron oxides (USPIO), with a hydrodynamic size less than 50 nm and superparamagnetic iron oxides (SPIO) particles, with hydrodynamic size between 50 and a few hundred nanometers. Magnetic nanoparticles are biodegradable, participating in the iron homeostasis in the body [22].

One important aspect of the use of magnetic nanoparticles in the delivery of drugs is linked to their magnetic capabilities. Under the local application of a magnet generating a strong magnetic field, magnetic nanoparticles tend to accumulate into a specific site. This property was evaluated in regenerative medicine for cardiovascular applications [25–27].

In the next section, we describe the preclinical uses of nanosystems in the delivery of thrombolytic drugs.

## Thrombolytic therapy using nanocarriers & microbubbles in preclinical development

The use of nano- and micro-carriers to deliver thrombolytic drugs shows several advantages such as protection from inhibitors present in the blood and concentration of drugs at the thrombus. The nanocarriers have a size between a few nanometers to a few hundred of nanometers. Microbubbles have a size between 2 and 8 µm and are composed of a shell made of phospholipid, polymer or albumin, and a core loaded with air or high molecular weight gas [28]. Only three thrombolytic drugs have been loaded into nano- and micro-carriers and tested *in vivo*. These aspects will be detailed in this section dedicated to preclinical development.

### Streptokinase

The history of thrombolytic therapy began in 1933, with the discovery, by Tillett and Garner, that certain strains of Streptococcus were able to dissolve fibrin clots [10,29]. SK is a single chain protein produced by different strains of streptococcal bacteria [30]. This protein has a molecular weight of 47 kDa and contains 414 amino acids. SK indirectly catalyses the activation of plasminogen [31] by binding with free circulating plasminogen and thus forming a complex that converts plasminogen to plasmin [9]. SK activates not only fibrin-bound plasminogen but also the free plasminogen inducing serious bleeding complications [30]. This protein shows a biphasic half-life, a first one rapid (16 min) and a second one longer (90 min). The first initial half-life is linked to the complexation of anti-SK antibodies while the second half-life is due to biological elimination of the protein. SK is inexpensive but shows an immunogenic effect and his activity is affected by the presence of antibodies [9,10].

#### Nanocarriers loaded with SK

In order to reduce side effects induced by SK (Streptase^®^) treatment, Leach *et al*. encapsulated the enzyme into naked liposomes and into a water-soluble double emulsion polymer (PEG and PVA). The authors noted some difficulties with the stability of the liposomes. Only 30% of the initial SK was entrapped within liposomes. The SK yield into polymeric porous particles was 82%. When injected into rabbits with autologous carotid artery thrombosis, the time necessary for reperfusion was significantly reduced: on average 7.3 min were necessary for SK loaded into polymers, 19.3 min for SK-liposome and 74.5 min for free SK [32]. Later, the same team tested these polymer-based nanoparticles in dogs with autologous coronary thrombus. The polymer-based nanoparticles showed a greater reduction in the time required to achieve reperfusion than free SK. In parallel, a reduction of infarct size and less hemorrhage were observed. These results were probably due to the enhanced transport of the encapsulated drug into the core of the thrombus. This accumulation was associated with the polymer dissolution and the release of protected SK [33,34].

Vaidya *et al*. obtained 18% of SK entrapment efficacy into liposomes. The SK was loaded into liposomes during hydration of the lipid film. The liposomes were moreover loaded with RGD (arginine-glycine-aspartic acid) peptide in order to target GPIIb/IIIa receptor expressed at the surface of the activated platelets in thrombi (Figure 4). The liposomes were injected into rats with carotid thrombosis generated by a human clot. Thirty minutes after treatment, a better thrombolytic activity was observed in rats receiving liposomes encapsulating SK compared with SK alone (28 vs 17%, respectively). This thrombolytic effect was explained by the protection of the SK simultaneous to the accumulation of targeted liposomes into clot [35].

Taken together these preclinical results show that loading the SK into nanocarriers protects it from premature inactivation in the blood and improves its accumulation into the clot leading to an enhanced thrombolysis.

### Urokinase

UK is a serine protease that activates plasminogen into plasmin thus degrading fibrin clots [36]. It is a protein with two polypeptide chains of 32 kDa and 54 kDa, respectively. Originally obtained from human urine, UK is now produced from human renal cell lines. Into whole blood, UK has a half-life from 15 to 20 min. High amounts are necessary to obtain a significant thrombolytic effect, inducing undesirable side effects such as hemorrhages [9]. Like SK, UK activates both the circulating and the fibrin-bound plasminogen [10].

#### Nanocarriers loaded with urokinase

Jin *et al*. prepared UK-loaded in water-soluble chitosan nanoparticles using an ionic cross-linking method. The final size was 236 nm with a drug encapsulation efficiency of 95%. They injected these nanocarriers into rabbits with jugular thrombosis obtained after administration of thrombin. An increased capacity of clot lysis was observed when compared with free UK. *In vivo*, free UK was metabolized rapidly with a half-life <20 min while the UK loaded inside the NPs showed a slow release rate [37].

Dextran-coated magnetic nanoparticles were used for covalent bioconjugation with UK via primary amine. The nanoparticles with 116-nm size were injected into rats with autologous carotid artery and left jugular vein thrombosis. When applying permanent magnets in the thrombus area, the nanoparticles were concentrated into the thrombotic site, showing a fivefold higher thrombolytic activity than free UK [38].

In an interesting approach, Marsh *et al*. developed perfluorocarbon (PFC) nanoparticles covalently coupled with UK. In order to target femoral thrombi generated in dogs, they coupled antifibrin antibody on the surface of the nanoparticles. When injected into the animals, the thrombus dissolution was higher in animals receiving PFC-UK loaded and antifibrin functionalized nanoparticles than in control animals receiving irrelevant IgG targeted UK PFC nanoparticles [39]. This thrombus specificity was achieved by fibrin monoclonal antibodies allowing an enhanced delivery of UK.

Mu *et al*. have successfully loaded UK and RGD to the surface of a microbubble-based ultrasound contrast agent. They observed an aggregation at the surface of femoral arterial thrombi in rabbits. However, the authors did not quantify the *in vivo* thrombolytic activity [40]. In rabbits with middle cerebral artery occlusion receiving UK and sulfur hexafluoride microbubbles, the addition of transcranial Doppler ultrasounds (2MHz) showed an enhancement of the recanalization rate compared with animals receiving only UK [41].

### Tissue plasminogen activator & its recombinant forms (tPA, rtPA)

tPA is encoded in humans by a gene on chromosome 8 and is produced by endothelial cells. tPA has two inhibitors, PAI-1 and PAI-2, which belong to the serpin superfamily. The tPA residues 296–304 are critical for the interaction with PAI-1. It shows a limited half-life of about 4–6 min [9]. Recombinant tPA (rtPA, Alteplase) is a serine protease with a molecular weight of 68 kDa. This recombinant form is produced by Chinese hamster ovary (CHO) cell lines by cDNA technology. This enzyme cleaves the Arg–Val bond, inducing the plasminogen conversion into plasmin [10].

#### Nanocarriers loaded with rtPA

Uesugi *et al*. have loaded rtPA (Cleactor^®^) on cationizated gelatin and PEG-grafted nanoparticles. When injected into rabbits with balloon injury of right femoral artery, the half-life of rtPA was three times enhanced due to its complexation with gelatin. In order to induce thrombolysis they applied ultrasound (1MHz, 0.75 W/cm^2^) for up for 60 min [24]. A complete recanalization was observed, which was probably linked to the dissociation of rtPA from gelatin and maybe to the effects of ultrasound on thrombus. Similar results were obtained on swine with acute myocardial infarction with a thrombotic occlusion of left coronary artery. A high recanalization rate was observed in 9 out of 10 swines when receiving nanoparticles, whereas only 1 out of 10 showed complete recanalization in a group with rtPA alone [42].

Zhou *et al*. developed PLGA nanocarriers loaded with rtPA (Alteplase). In order to enhance the accumulation in a rat model of abdominal aortic thrombi induced using ferric chloride, they covered the nanoparticles with a chitosan shell and targeted it with cyclic RGD peptide via a carbodiimide bond. The rtPA loaded into the nanoparticles showed a prolonged half-life. The authors reported some limitations such as the encapsulation efficacy of the rtPA (between 54 and 64%) simultaneously to a partial loss of rtPA activity [43].

Magnetic nanoparticles coated with polyacrylic acid (PAA) were covalently coupled with an amine group of rtPA (Alteplase). Magnetic nanoparticles show the advantage of being made of a magnetite (Fe_3_O_4_) core that responds to an external magnetic field with superparamagnetic properties. The nanoparticles were concentrated into a thrombus under external magnetic guidance after injection into rats with iliac artery embolisms (Figure 5). The blood flow restoration was observed 75 min later with an equivalent of 0.2 mg/kg of rtPA coupled with magnetic nanoparticles. With a dose of 1 mg/kg, the blood flow restoration was obtained within only 30 min [44]. The same team later developed magnetic nanoparticles coated with a shell of poly (aniline-co-N-[1-one-butyric acid] aniline) and loaded with rtPA. With this strategy the amount of rtPA loaded was 50% higher than the amount loaded previously into PAA-coated nanoparticles. After injection into rats with iliac embolisms, the nanoparticles were guided towards the clots using an external magnetic field [45]. rtPA (Alteplase) was covalently immobilized on chitosan-coated magnetic nanoparticles and injected into rats with iliac artery embolisms. The administration of rtPA loaded on chitosan magnetic nanoparticles associated with a magnetic guidance resulted in 70–80% blood flow recovery with a fivefold lower dosage, 0.2 mg/kg compared with 1 mg/kg required for thrombolysis with free rtPA [23].

These nanocarriers reduce the effective dose of active principle injected but also increase the local concentration and prevent side effects. A limitation of this method could be linked to the fact that the external magnetic field decreases with the tissue depth, and thus makes it difficult to target a site deeper in the body. Kempe *et al*., implanted ferromagnetic stents for the treatment of in-stent thrombosis. In their approach, they used PEGylated magnetic nanoparticles loaded with rtPA (Alteplase). The nanoparticles were injected in pigs with ferromagnetic stents in their coronary artery. The restoration of blood flow with rtPA loaded nanoparticles was reached with a lower amount of drug compared with free rtPA [46].

The McCarthy team coupled rtPA (Alteplase) to cross-linked dextran-coated iron oxide nanoparticles (CLIO). In order to increase the distance between the fibrinolytic drug and the particles and to minimize the steric interactions they added a PEG spacer moiety. The nanoparticles were moreover functionalized by covalent grafting of activated factor XIII (FXIIIa) via peptide affinity ligands. They injected these targeted nanoparticles (CLIO-FXIII-PEG-rtPA) into mice with pulmonary embolisms obtained with human clots. This choice was based on the facts that the murine plasminogen system is tenfold less sensitive to human tPA. The authors concluded that the targeted nanoagent displayed similar thrombolytic potential as free tPA [47]. These studies showed that magnetic nanoparticles have a potential for local delivery of thrombolytic drugs either by an external magnetic field or with peptides.

The loading of rtPA (Alteplase) into liposomes obtained using the freeze-thawing preparation method, did not alter the fibrinolytic activity of the drug. The rtPA loaded into PEGylated liposomes showed 21-fold prolonged circulation time compared with free rtPA [17]. Absar *et al*. developed liposomes, either coated with PEG or not, and decorated with a peptide sequence of fibrinogen gamma-chain targeting GPIIb/IIIa. Entrapment efficacy of rtPA (Alteplase) varied from 12 to 26% for non-PEGylated liposomes and from 36 to 52% for PEGylated liposomes. These nanoparticles were injected into rats with inferior vena cava thrombosis induced with a FeCl_3_ solution. An enhancement of 35% of thrombolytic activity was observed for rtPA-loaded into targeted liposomes when compared with native rtPA. Tested *ex vivo* on human clot, liposomal rtPA showed a slightly lower activity compared with native rtPA, this being probably linked to the incomplete release of the drug from liposomes [48].

An interesting approach to enhance the thrombolysis is the use of ultrasound as adjuvant therapy. This strategy was developed with echogenic liposomes as well as with microbubbles. Echogenic liposomes (ELIP) are composed of a phospholipid bilayer enclosing both gas and liquid. The advantage of this system is the follow-up by echography of thrombus evolution before, during and after thrombolysis. Moreover, ELIP are not only ultrasound contrast agents but also potential vectors for thrombolysis. Laing *et al*. developed ELIP loaded with rtPA (Alteplase) in the core (15%) or associated with the phospholipid bilayer (35%). The rtPA solution was loaded into liposomes during the hydratation of lipid film. ELIP loaded with rtPA were injected into rabbits with abdominal aortic thrombi exposed to ultrasound (pulsed ultrasound 5.7 MHz for 2 min). The degree of recanalization determined by Doppler flow measurements, showed that ELIP charged with rtPA had similar efficacy to free rtPA for thrombus dissolution *in vivo*. Injection of saline (control), empty ELIP, or empty ELIP associated with ultrasound did not show any thrombolytic effect [49]. The recanalization rate was variable in the absence of ultrasound, showing that ultrasound therapy enhance thrombolytic effect [50]. This enhancement could be explained by acoustic cavitation, thermal effects or microstreaming (Figure 6) [51–53].

In order to enhance accumulation into thrombus, Hagisawa *et al*. developed perfluorocarbon based echogenic liposomes targeted with a RGD peptide loaded or not with rt-PA (Monteplase). They injected them intravenously into rabbits with thrombus in ilio-femoral arteries and applied ultrasound. A higher recanalization rate (nine out of ten rabbits) was observed when ultrasounds were applied, compared with that of animals receiving nontargeted liposomes (two out of ten rabbits) or rtPA monotherapy (four out of ten rabbits) [54].

Microbubbles with a size from 2 to 8 µm represent another class of lipid based carriers used in thrombolytic strategies. They are composed of a shell made of phospholipids, polymers or albumin, and a core of air or high molecular weight gas [28]. The effect of sulfur hexafluoride lipid based microbubbles associated with ultrasound was compared with intravenous administration of rtPA (10 mg/kg) into rats with acute cerebral ischemia obtained after autologous thrombus injection into carotid artery. The two modalities of treatment showed equivalent result in the restoration of blood flow [55].

Nedelmann *et al*. showed on rats with filament occlusion of the right middle cerebral artery that the association of rtPA (Alteplase) with microbubbles and ultrasounds completely restored the blood flow, while rtPA alone partially improved hemispheric perfusion [56]. In a rabbit model of embolic stroke obtained with a clot from a donor rabbit, the combination of microbubbles with rtPA and pulsed ultrasound (1MHz) showed a good recanalization rate [57]. These studies demonstrated that ultrasound could be associated with microbubbles and/or thrombolytic drugs to enhance the recanalization effect.

## Microbubbles associated with thrombolytic drugs and/or ultrasound for clinical applications

Microbubbles are the only platform tested in the clinic for thrombolytic therapy. In clinical trials, they were associated or not with ultrasound and showed encouraging results. Molina *et al*. included 111 patients with middle cerebral artery occlusions. The 38 patients who received galactose-based microbubbles, rtPA and 2 MHz ultrasound pulse showed a better recanalization rate than the other 73 patients who received either rtPA and ultrasound or rtPA only [58,59]. Later, they associated in another clinical trial, perflutren lipid (MRX-801) microbubbles with rtPA and ultrasound and obtained 67% of recanalization rate compared with 46% for the group receiving only rtPA [60]. Alexandrov *et al*. showed that half of patients receiving rtPA, 2 MHz continuous TCD monitoring and perflutren-lipid based microbubbles demonstrated a complete recanalization rate (six patients out of 12) while none of the patients receiving only rtPA reached a complete recanalization [61]. Microbubbles based on phospholipids encapsulating sulfur hexafluoride combined with transcranial ultrasound and rtPA showed a better recanalization rate compared with patients receiving rtPA and ultrasound [62].

Another clinical trial was made by Pagola *et al*. in patients with stroke with basilar artery occlusion. All the 20 patients received intravenous rtPA, ultrasound and galactose based microbubbles. At 24 h, only 50% of patients showed a progressive recanalization while the other 50% did not show any recanalization [63]. Rubiera *et al*. showed that the recanalization was similar in patients receiving either galactose-based microbubbles or phospholipid-based microbubbles encapsulating sulfur-hexafluoride, and rtPA associated with 2 MHz transcranial Doppler [64].

One possible explanation of thrombolysis enhancement when microbubbles and ultrasound are associated with rtPA, is the mechanical damage induced by the streaming of microbubbles at the surface of the thrombus allowing a higher diffusion of rtPA. This opens new perspectives for clinical thrombolytic therapy with the aim to reduce the dose and, therefore, hemorrhagic side effects.

## Discussion & conclusion

Nano- and micro-carriers are able to replace the systemic therapy of whole body with a local therapy reducing significantly undesirable side effects. Although ischemic heart and stroke caused by thrombosis are the main causes of death in the world, there is no existing nanocarrier loaded with thrombolytic drug in clinic. Currently, only galactose or lipid-based microbubbles associated to ultrasound with or without rtPA systemic injection have been evaluated in clinical trials and showed an enhancement of thrombolytic effect. In other pathologies such as cancer or infectious diseases, different therapeutic drugs loaded to nanocarriers have already reached the market. This gap could be partly explained by the distinctive features of thrombosis and of the thrombolytic drugs.

In the case of heart ischemia or stroke it is necessary to act rapidly within the first minutes or hours (<4.30 h) after the appearance of the symptoms. Thrombolytic drugs administered by systemic way are quickly inactivated by inhibitors in blood. Nanocarriers have the advantage to protect the drug, enhancing its half-life in the blood. Encouraging results were thus obtained on preclinical models with lipid or polymer systems loaded with thrombolytic drugs.

The drugs loaded into nanocarriers must keep their therapeutic capacities. The therapeutic drugs are either charged on the surface of the nanoparticles, as for magnetic nanoparticles, or loaded inside nanocarriers, which is the case of polymer or lipid based systems. Another critical point is linked to the amount of drug loaded into nanocarriers. The loading capacity must be maximal in order to reduce the injected quantity of carriers. The preclinical results obtained with liposomes showed a low entrapment efficacy, while a better efficacy was usually obtained with polymer based nanoparticles.

An interesting aspect linked to the use of nanocarriers loaded with therapeutic drugs is the capacity to achieve an active targeting of the thrombus, improving the therapeutic efficacy. This was for instance possible by using targeting peptides on the surface of nanocarriers. Another modality of targeting tested on preclinical models was based on the use of magnetic nanoparticles. Although encouraging results were obtained in small animals, this method seems difficult to apply in patients because of the deep localization of the thrombus. Maybe, the use of large and ultrastrong magnets would be a possibility to overcome this limitation in the near future.

For all systems, a common limitation is linked to the difficulty to obtain a controlled release. The use of ultrasound can partially control the release into thrombus of therapeutics loaded into echogenic liposomes. However, the ultrasound have a limited penetration into the body, and the use of high frequencies could induce blood vessel lesions. Again, improvements of ultrasound equipment may increase its efficacy.

Another important aspect is linked to the thrombus itself in preclinical studies. Some preclinical models are made on animals with an autologous thrombus. However, the affinity of thrombolytics used in clinics for the animal clot is not similar to the affinity for the human one. The use of animal models obtained with a human clot would thus be more relevant. The aging of the used thrombus is also relevant.

In conclusion, more in depth preclinical research and developments are necessary to improve the targeted delivery of thrombolytic drugs before their translation into clinical practice.

## Future perspective

Nanomedicine offers an opportunity and a challenge in the management of a targeted thrombolytic therapy. There are some specific features to take into account for thrombus therapy using nanocarriers. The nanocarrier should load a maximum amount of drug while keeping the thrombolytic efficacy, protect the drug from enzymatic degradation and ensure a rapid release in the thrombus. A balance between all these aspects is mandatory for a better patient care. The development of new materials for nanocarriers and the discovery of new thrombolytic drugs are also considered in the development of nanothrombolysis.

**Table 1.** **Examples of clinically used thrombolytic drugs.**

**Thrombolytic agent**	**Abbreviation**	**Molecular weight (kDa)**	**Indication**	**US FDA status**	**Structure (domains)**	**Half-life (min)**	**Fibrino selective**	**Elimination**
Urokinase (Abbokinase^®^, Abbot Laboratories, TX, USA)	UK	2 polypeptides chains (32/54)	AMI, PE	Approved	P/K/EGF	15–20	No	Kidney
Streptokinase (Streptase)	SK	47	AMI, PE, DVT, PAO	Approved	α, β, γ	10–16	No	Kidney
Alteplase (Activase^®^, Genentech, CA, USA; Actilyse^®^, Boehringer Ingelheim, Germany)	tPA, rtPA	68	AMI, PE, IS	Approved	F/EGF/K1/K2/S	4–6	Yes	Liver
Tenecteplase (TNKase^®^, Genentech, USA; Metalyse^®^, Boehringer Ingelheim, Germany)	TNK-tPA	70	AMI	Approved	F/EGF/K1	20	Yes	Kidney
Reteplase (Retavase^®^, Chiesi Farmaceutici S.p.A, Italy; Rapilysin^®^, Actavis, NJ, USA)	rPA	40	AMI, DVT, PAO	Phase II	K2/SP	18	Yes	Kidney
Staphylokinase	SAK	16.5	AMI	Phase II	2 chains: a, b	6	Yes	Liver
Lanoteplase	nPA	53.5	AMI	Phase II	F/K1/K2/SP	37	Yes	Liver
Desmoteplase	batPA	52	IS	Phase III	F/EGF/K1/SP	240	Yes	Liver

AMI: Acute myocardial infarction; DVT: Deep-vein thrombosis; EGF: EGF domain; F: Finger domain; IS: Ischemic stroke; K1: Kringle 1 domain; K2: Kringle 2 domain; PAO: Peripheral arterial occlusion; PE: Pulmonary embolism; SP: Serine protease domain.

**Executive summary**

Heart ischemia and stroke are the main causes of death in the world.
Tissue plasminogen activator but also urokinase and streptokinase are the thrombolytic drugs used in clinic. These drugs are inactivated by circulating inhibitors. Therefore, high amounts are injected to patients in order to obtain a therapeutic effect but with a risk of undesirable side effects such as intracranial hemorrhages.
Polymer-, lipid- or magnetic-based nano- and micro-carriers are able to deliver thrombolytic drugs according to their size and structure.
In preclinical studies, nanoparticles loaded with either urokinase, streptokinase or recombinant tissue plasminogen activator (rtPA) have been evaluated. As compared with free drug, a lower amount of drug loaded into nanoparticles was necessary to induce a thrombolytic effect. In parallel, a reduction of intracranial hemorrhages was observed.
In clinical trials, only galactose and lipid-based microbubbles were evaluated. They were associated to ultrasound and/or rtPA. Enhancement of thrombolytic efficacy was observed with microbubbles associated to ultrasound and rtPA. However, further validation is necessary before a daily clinical use.
In conclusion, loading thrombolytic drugs into nanocarriers opens new perspectives for thrombotic diseases therapy.

## References

[1] Go AS, Mozaffarian D, Roger VL (2013). Heart disease and stroke statistics – 2013 update: a report from the american heart association. *Circulation*.

[2] Hilgendorf I, Swirski FK, Robbins CS (2015). Monocyte fate in atherosclerosis. *Arterioscler. Thromb. Vasc. Biol.*.

[3] Falk E (2006). Pathogenesis of atherosclerosis. *J. Am. Coll. Cardiol.*.

[4] Fuster V, Fayad ZA, Moreno PR, Poon M, Corti R, Badimon JJ (2005). Atherothrombosis and high-risk plaque: part ii: approaches by noninvasive computed tomographic/magnetic resonance imaging. *J. Am. Coll. Cardiol.*.

[5] Bivard A, Lin L, Parsonsb MW (2013). Review of stroke thrombolytics. *J. Stroke*.

[6] Furie B, Furie BC (2008). Mechanisms of thrombus formation. *N. Engl. J. Med.*.

[7] Mackman N (2008). Triggers, targets and treatments for thrombosis. *Nature*.

[8] Mazighi M, Serfaty JM, Labreuche J (2009). Comparison of intravenous alteplase with a combined intravenous-endovascular approach in patients with stroke and confirmed arterial occlusion (recanalise study): a prospective cohort study. *Lancet Neurol.*.

[9] Baruah DB, Dash RN, Chaudhari MR, Kadam SS (2006). Plasminogen activators: a comparison. *Vascul. Pharmacol.*.

[10] Kotb E (2014). The biotechnological potential of fibrinolytic enzymes in the dissolution of endogenous blood thrombi. *Biotechnol. Prog.*.

[11] Van De Craen B, Declerck PJ, Gils A (2012). The biochemistry, physiology and pathological roles of pai-1 and the requirements for pai-1 inhibition *in vivo*. *Thromb. Res.*.

[12] Fortenberry YM (2013). Plasminogen activator inhibitor-1 inhibitors: a patent review (2006–present). *Expert Opin. Ther. Pat.*.

[13] Copin JC, Bengualid DJ, Da Silva RF, Kargiotis O, Schaller K, Gasche Y (2011). Recombinant tissue plasminogen activator induces blood–brain barrier breakdown by a matrix metalloproteinase-9-independent pathway after transient focal cerebral ischemia in mouse. *Eur. J. Neurosci.*.

[14] Klink A, Hyafil F, Rudd J (2011). Diagnostic and therapeutic strategies for small abdominal aortic aneurysms. *Nat. Rev. Cardiol.*.

[15] Silva AK, Letourneur D, Chauvierre C (2014). Polysaccharide nanosystems for future progress in cardiovascular pathologies. *Theranostics*.

[16] Ricklin D, Hajishengallis G, Yang K, Lambris JD (2010). Complement: a key system for immune surveillance and homeostasis. *Nat. Immunol.*.

[17] Kim JY, Kim JK, Park JS, Byun Y, Kim CK (2009). The use of pegylated liposomes to prolong circulation lifetimes of tissue plasminogen activator. *Biomaterials*.

[18] Koshkaryev A, Sawant R, Deshpande M, Torchilin V (2013). Immunoconjugates and long circulating systems: Origins, current state of the art and future directions. *Adv. Drug. Deliv. Rev.*.

[19] Rabanel JM, Hildgen P, Banquy X (2014). Assessment of peg on polymeric particles surface, a key step in drug carrier translation. *J. Control. Release*.

[20] Bowey K, Tanguay JF, Tabrizian M (2012). Liposome technology for cardiovascular disease treatment and diagnosis. *Expert Opin. Drug Deliv.*.

[21] Ruiz-Esparza GU, Flores-Arredondo JH, Segura-Ibarra V (2013). The physiology of cardiovascular disease and innovative liposomal platforms for therapy. *Int. J. Nanomedicine*.

[22] Tassa C, Shaw SY, Weissleder R (2011). Dextran-coated iron oxide nanoparticles: a versatile platform for targeted molecular imaging, molecular diagnostics, and therapy. *Acc. Chem. Res.*.

[23] Chen JP, Yang PC, Ma YH, Wu T (2011). Characterization of chitosan magnetic nanoparticles for *in situ* delivery of tissue plasminogen activator. *Carbohydrate Polymers*.

[24] Uesugi Y, Kawata H, Jo J, Saito Y, Tabata Y (2010). An ultrasound-responsive nano delivery system of tissue-type plasminogen activator for thrombolytic therapy. *J. Control. Release*.

[25] Robert D, Fayol D, Le Visage C (2010). Magnetic micro-manipulations to probe the local physical properties of porous scaffolds and to confine stem cells. *Biomaterials*.

[26] Cheng K, Li TS, Malliaras K, Davis DR, Zhang Y, Marban E (2010). Magnetic targeting enhances engraftment and functional benefit of iron-labeled cardiosphere-derived cells in myocardial infarction. *Circ. Res.*.

[27] Silva AK, Luciani N, Gazeau F (2015). Combining magnetic nanoparticles with cell derived microvesicles for drug loading and targeting. *Nanomedicine*.

[28] De Saint Victor M, Crake C, Coussios CC, Stride E (2014). Properties, characteristics and applications of microbubbles for sonothrombolysis. *Expert Opin. Drug Deliv.*.

[29] Collen D, Lijnen HR (2005). Thrombolytic agents. *Thromb. Haemost.*.

[30] Butcher K, Shuaib A, Saver J (2013). Thrombolysis in the developing world: is there a role for streptokinase?. *Int. J. Stroke*.

[31] Kunamneni A, Abdelghani TT, Ellaiah P (2007). Streptokinase – the drug of choice for thrombolytic therapy. *J. Thromb. Thrombolysis*.

[32] Leach JK, O'Rear EA, Patterson E, Miao Y, Johnson AE (2003). Accelerated thrombolysis in a rabbit model of carotid artery thrombosis with liposome-encapsulated and microencapsulated streptokinase. *Thromb. Haemost.*.

[33] Leach JK, Patterson E, O'Rear EA (2004). Encapsulation of a plasminogen activator speeds reperfusion, lessens infarct and reduces blood loss in a canine model of coronary artery thrombosis. *Thromb. Haemost.*.

[34] Leach JK, Patterson E, O'Rear EA (2004). Distributed intraclot thrombolysis: mechanism of accelerated thrombolysis with encapsulated plasminogen activators. *J. Thromb. Haemost.*.

[35] Vaidya B, Agrawal GP, Vyas SP (2011). Platelets directed liposomes for the delivery of streptokinase: development and characterization. *Eur. J. Pharm. Sci.*.

[36] Kunamneni A, Ravuri BD, Saisha V, Ellaiah P, Prabhakhar T (2008). Urokinase – a very popular cardiovascular agent. *Recent Pat. Cardiovasc. Drug Discov.*.

[37] Jin HJ, Zhang H, Sun ML, Zhang BG, Zhang JW (2013). Urokinase-coated chitosan nanoparticles for thrombolytic therapy: preparation and pharmacodynamics *in vivo*. *J. Thromb. Thrombolysis.*.

[38] Bi F, Zhang J, Su Y, Tang YC, Liu JN (2009). Chemical conjugation of urokinase to magnetic nanoparticles for targeted thrombolysis. *Biomaterials*.

[39] Marsh JN, Hu G, Scott MJ (2011). A fibrin-specific thrombolytic nanomedicine approach to acute ischemic stroke. *Nanomedicine (Lond.)*.

[40] Mu Y, Li L, Ayoufu G (2009). Experimental study of the preparation of targeted microbubble contrast agents carrying urokinase and rgds. *Ultrasonics*.

[41] Liu WS, Huang ZZ, Wang XW, Zhou J (2012). Effects of microbubbles on transcranial doppler ultrasound-assisted intracranial urokinase thrombolysis. *Thromb. Res.*.

[42] Kawata H, Uesugi Y, Soeda T (2012). A new drug delivery system for intravenous coronary thrombolysis with thrombus targeting and stealth activity recoverable by ultrasound. *J. Am. Coll. Cardiol.*.

[43] Zhou J, Guo D, Zhang Y, Wu W, Ran H, Wang Z (2014). Construction and evaluation of Fe(_3_)O(_4_)-based PLGA nanoparticles carrying rtPA used in the detection of thrombosis and in targeted thrombolysis. *ACS. Appl. Mater. Interfaces*.

[44] Ma YH, Wu SY, Wu T, Chang YJ, Hua MY, Chen JP (2009). Magnetically targeted thrombolysis with recombinant tissue plasminogen activator bound to polyacrylic acid-coated nanoparticles. *Biomaterials*.

[45] Yang HW, Hua MY, Lin KJ (2012). Bioconjugation of recombinant tissue plasminogen activator to magnetic nanocarriers for targeted thrombolysis. *Int. J. Nanomedicine*.

[46] Kempe M, Kempe H, Snowball I (2010). The use of magnetite nanoparticles for implant-assisted magnetic drug targeting in thrombolytic therapy. *Biomaterials*.

[47] Mccarthy JR, Sazonova IY, Erdem SS (2012). Multifunctional nanoagent for thrombus-targeted fibrinolytic therapy. *Nanomedicine (Lond.)*.

[48] Absar S, Nahar K, Kwon YM, Ahsan F (2013). Thrombus-targeted nanocarrier attenuates bleeding complications associated with conventional thrombolytic therapy. *Pharm. Res.*.

[49] Laing ST, Moody M, Smulevitz B (2011). Ultrasound-enhanced thrombolytic effect of tissue plasminogen activator-loaded echogenic liposomes in an *in vivo* rabbit aorta thrombus model – brief report. *Arterioscler. Thromb. Vasc. Biol.*.

[50] Laing ST, Moody MR, Kim H (2012). Thrombolytic efficacy of tissue plasminogen activator-loaded echogenic liposomes in a rabbit thrombus model. *Thromb. Res.*.

[51] Brujan EA (2009). Cardiovascular cavitation. *Med. Eng. Phys.*.

[52] Unger E, Porter T, Lindner J, Grayburn P (2014). Cardiovascular drug delivery with ultrasound and microbubbles. *Adv. Drug Deliv. Rev.*.

[53] Chen X, Leeman JE, Wang J, Pacella JJ, Villanueva FS (2014). New insights into mechanisms of sonothrombolysis using ultra-high-speed imaging. *Ultrasound Med. Biol.*.

[54] Hagisawa K, Nishioka T, Suzuki R (2013). Thrombus-targeted perfluorocarbon-containing liposomal bubbles for enhancement of ultrasonic thrombolysis: *in vitro* and *in vivo* study. *J. Thromb. Haemost.*.

[55] Moumouh A, Barentin L, Tranquart F, Serrierre S, Bonnaud I, Tasu JP (2010). Fibrinolytic effects of transparietal ultrasound associated with intravenous infusion of an ultrasound contrast agent: study of a rat model of acute cerebral stroke. *Ultrasound Med. Biol.*.

[56] Nedelmann M, Ritschel N, Doenges S (2010). Combined contrast-enhanced ultrasound and rt-pa treatment is safe and improves impaired microcirculation after reperfusion of middle cerebral artery occlusion. *J. Cereb. Blood Flow Metab.*.

[57] Brown AT, Flores R, Hamilton E, Roberson PK, Borrelli MJ, Culp WC (2011). Microbubbles improve sonothrombolysis *in vitro* and decrease hemorrhage *in vivo* in a rabbit stroke model. *Invest. Radiol.*.

[58] Tsivgoulis G, Culp WC, Alexandrov AV (2008). Ultrasound enhanced thrombolysis in acute arterial ischemia. *Ultrasonics*.

[59] Molina CA, Ribo M, Rubiera M (2006). Microbubble administration accelerates clot lysis during continuous 2-MHz ultrasound monitoring in stroke patients treated with intravenous tissue plasminogen activator. *Stroke*.

[60] Molina CA, Barreto AD, Tsivgoulis G (2009). Transcranial ultrasound in clinical sonothrombolysis (tucson) trial. *Ann. Neurol.*.

[61] Alexandrov AV, Mikulik R, Ribo M (2008). A pilot randomized clinical safety study of sonothrombolysis augmentation with ultrasound-activated perflutren–lipid microspheres for acute ischemic stroke. *Stroke*.

[62] Perren F, Loulidi J, Poglia D, Landis T, Sztajzel R (2008). Microbubble potentiated transcranial duplex ultrasound enhances iv thrombolysis in acute stroke. *J. Thromb. Thrombolysis*.

[63] Pagola J, Ribo M, Alvarez-Sabin J, Lange M, Rubiera M, Molina CA (2007). Timing of recanalization after microbubble-enhanced intravenous thrombolysis in basilar artery occlusion. *Stroke*.

[64] Rubiera M, Ribo M, Delgado-Mederos R (2008). Do bubble characteristics affect recanalization in stroke patients treated with microbubble-enhanced sonothrombolysis?. *Ultrasound Med. Biol.*.

